# The Chemical Characterization of Nigerian Propolis samples and Their Activity Against *Trypanosoma brucei*

**DOI:** 10.1038/s41598-017-01038-2

**Published:** 2017-04-19

**Authors:** Ruwida Omar, John O. Igoli, Tong Zhang, Alexander I. Gray, Godwin U. Ebiloma, Carol J. Clements, James Fearnley, RuAngeli Edrada Ebel, Tim Paget, Harry P. de Koning, David G. Watson

**Affiliations:** 1 University of Strathclyde, Strathclyde Institute of Pharmacy and Biomedical Science, 161 Cathedral Street, Glasgow, G4 0RE UK; 2 Phytochemistry Research Group, Department of Chemistry, University of Agriculture, Makurdi, Nigeria; 3 Wolfson Wohl Cancer Research Centre, Institute of Cancer Sciences, University of Glasgow, Switchback Road, Glasgow, G61 1QH UK; 4 Institute of Infection, Immunity and Inflammation, College of Medical, Veterinary and Life Sciences, University of Glasgow, Glasgow, G12 8TA UK; 5 BeeVital, Whitby, North Yorkshire YO22 5JR UK; 6 Department of Pharmacy, Health and Well-being, University of Sunderland, Wharncliffe Street, Sunderland, SR1 3SD UK

## Abstract

Profiling of extracts from twelve propolis samples collected from eight regions in Nigeria was carried out using high performance liquid chromatography (LC) coupled with evaporative light scattering (ELSD), ultraviolet detection (UV) and mass spectrometry (MS), gas chromatography mass spectrometry (GC-MS) and nuclear magnetic resonance spectroscopy (NMR). Principal component analysis (PCA) of the processed LC-MS data demonstrated the varying chemical composition of the samples. Most of the samples were active against *Trypanosoma b*. *brucei* with the highest activity being in the samples from Southern Nigeria. The more active samples were fractionated in order to isolate the component(s) responsible for their activity using medium pressure liquid chromatography (MPLC). Three xanthones, 1,3,7-trihydroxy-2,8-di-(3-methylbut-2-enyl)xanthone, 1,3,7-trihydroxy-4,8-di-(3-methylbut-2-enyl)xanthone a previously undescribed xanthone and three triterpenes: ambonic acid, mangiferonic acid and a mixture of α-amyrin with mangiferonic acid (1:3) were isolated and characterised by NMR and LC-MS. These compounds all displayed strong inhibitory activity against *T*.*b*. *brucei* but none of them had higher activity than the crude extracts. Partial least squares (PLS) modelling of the anti-trypanosomal activity of the sample extracts using the LC-MS data indicated that high activity in the extracts, as judged from LCMS^2^ data, could be correlated to denticulatain isomers in the extracts.

## Introduction

Propolis is an important resinous apicultural product that bees collect from plants in order to seal up gaps in their hives and they also use it as an anti-infective agent to prevent infection of the hive by microorganisms^1^. A wide range of medicinal properties have been attributed to propolis, including antimicrobial, anti-hepatotoxic, anti-inflammatory, immunostimulant, anti-oxidative and anticancer properties, and there is an increasing interest in it as a source of new drugs^2^. It has a complex chemical composition, which depends on the local flora at the site of collection as well as the season of collection, and this makes it difficult and challenging to standardise^3^. Chemometric methods are being increasingly applied in order to standardise complex samples^4^ and have recently been applied to MS data obtained from propolis^5^. Since propolis consists of a wide range of organic compounds of varying polarity and ionisability, complementary techniques have been used in parallel to get a complete profile. In our previous work we examined propolis collected from many parts of Africa using a range of analytical methods^5^ and observed that there was no clear geographic delineation for the classification of these African propolis samples. One sample from Rivers State, Nigeria stood out as having a unique chemical composition and strong anti-trypanosomal activity and we went on to isolate ten compounds from this sample, which all displayed some degree of anti-trypanosomal activity^6^. Recently we carried out profiling and anti-protozoal testing of propolis samples from different regions in Libya^7^ which had a diverse chemical composition dependent on exact geographical origin. In this current paper we report the profiling and testing of propolis samples from different regions within Nigeria, which are considered to be within a wet savannah environment. Besides chemical characterization of these samples, we focused on activity against one particular infective organism which thrives in this environment and that is the protozoan parasite that is the causative agent of African Sleeping Sickness, *Trypanosoma brucei*. *Trypanosma* species are a health threat to cattle grazing in the savannah areas, as well as to humans. The existing drugs for treating either the human^8^ or veterinary disease^9^ are old, in many cases quite toxic, and often ineffective because of drug resistance^10^. Moreover, the rate of drug development against this disease is very slow. The vector for *T*. *brucei* is the tsetse fly, where the parasite initially colonises its gastro-intestinal tract. Members of the protozoal genus *Crithidia*, which are very closely related to *T*. *brucei*, having a similar life cycle and metabolism, similarly infect bees^11^ and have been mentioned as a possible cause of winter colony collapse in Europe^12^.

## Results and Discussion

### The Origin of the Nigerian Propolis Samples

**Figure 1:**
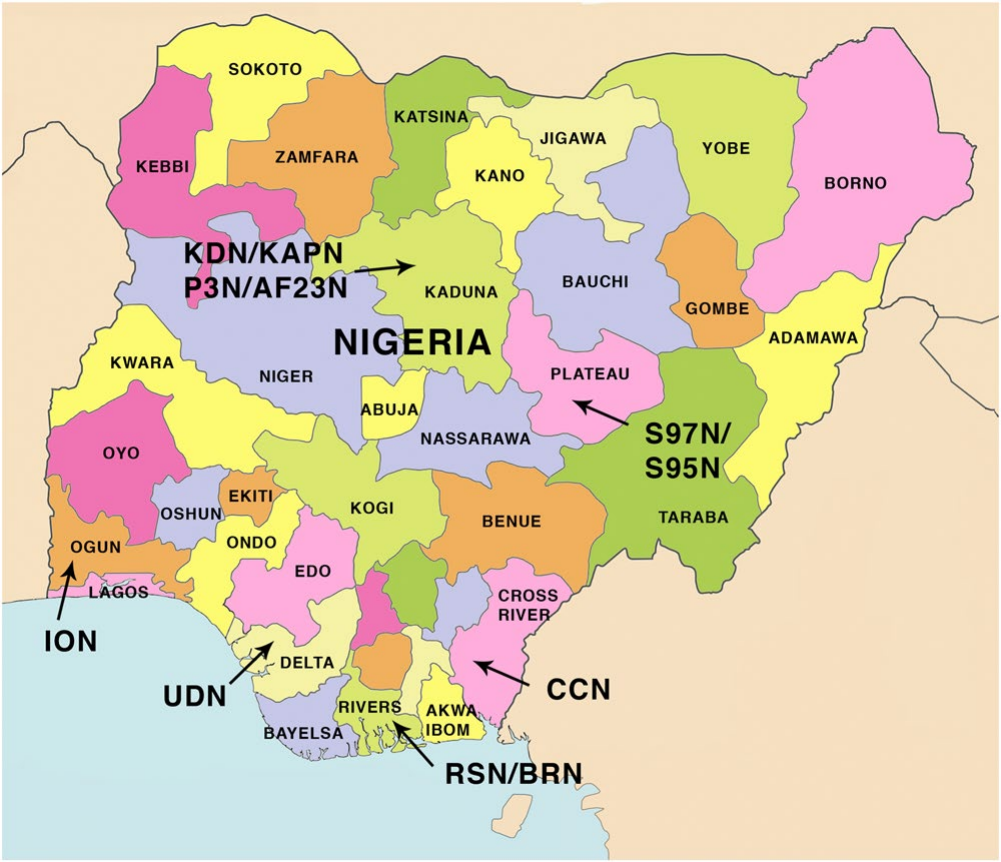
Map showing the locations of the collection of the Nigerian propolis samples.

Figure 1 and Table 1 show details of the origins of the Nigerian propolis samples.

**Table 1:** Codes, collection area and percentage of yield of ethanol extract (EEP) for the samples.

Code	Origin (town/State)	Collection date	% Yield (from 50 mg)
RSN and BRN	Bonny/River State Nigeria 4°26′ N 7°10′E	2003 and July 2013	76, 70.6
KAPN and P3N KKN	Kaduna/Kaduna State Nigeria 10°31′N 7°26′E	Unknown, October 2007, July 2013	73.6, 40 53.3
S97N	Jos, Plateau State Nigeria 9°56′N 8°53′E	February 2005	51
ION	Ijebu-Ode, Ogun State 6°49′N 3°55′E	July 2013	60.2
UDN	Ugelli, Delta State 5°30′N 5°59′E	July 2013	38.9
CCN	Calabar, Cross River State 4°57′ 8°19′E	July 2013	42.4
S95N	Jos Plateau State 9°56′N 8°53′E	February 2005	73
AF23N	Zaria, Kaduna State 11°04′N 7°42′E	NA	58.4
S96N	Unknown location in Nigeria	February 2005	52.4

### Bioassay of crude extracts from Nigerian Propolis

The crude ethanolic extracts from the Nigerian propolis samples were tested against *T*. *brucei* and *C*. *fasciculata*. The highest activities were in the samples from Southern Nigeria but all samples displayed some degree of activity apart from sample AF23N (Table 2). The activity against *C*. *fasciculata* supports the idea that the bees may collect propolis to protect from infection by *Crithidia* species which are known to be pathogens of bees^12^ and are quite closely related to *T*. *brucei*. Indeed there was a surprisingly good correlation between the EC_50_ values for the *T*. *brucei* control strain and for *Crithidia* (see Supplementary Figure S1), which further supports the idea that propolis provides a natural defence against *Crithidia* and could be exploited for treatments against other trypanosomatids. Moreover, the extracts were active against both the standard strain of *T*. *brucei* and two multi-drug resistant clones that displayed a high degree of resistance against pentamidine. It has been previously shown that clone B48, in particular, has very high levels of resistance to all diamidine drugs including pentamidine and diminazene aceturate, as well as the entire class of melaminophenyl arsenical drugs that includes melarsoprol and cymelarsan^13^ – i.e. most of the first-line human and veterinary treatments. Although slight differences (less than 2-fold sensitisation or resistance) were observed between the control and resistant strains these were very minor compared to the 225-fold or 31-fold resistance to pentamidine observed in parallel with clones B48 and aqp2/aqp3 null, respectively. The crude ethanol extracts were all complex mixtures and either individual components in the mixtures or isolated components might display higher activity.

**Table 2:** Bioassay of extracts from Nigerian propolis against *T*. *brucei* and *C*. *fasciculata*.

	*T*. *brucei* s427		*T*. *brucei* B48				*T*. *brucei* aqp2/aqp3 null				*C*. *fasciculata* (ATCC 50083)	
Sample	EC_50_^1^	SEM	EC_50_*	SEM	RF	ttest^3^	EC_50_*	SEM	RF	ttest^3^	EC_50_^2^	SEM
S95N	17.3	0.24	29.3	1.46	1.69	<0.01	17.9	0.12	1.03	0.10	21.3	1.6
S96N	23.4	0.77	31.4	1.09	1.34	<0.01	23.8	0.56	1.02	0.68	37.5	2
S97N	15.5	0.20	18.2	1.81	1.18	0.21	13.5	0.27	0.87	<0.001	ND	ND
P3N	32.6	0.33	47.2	3.91	1.45	0.02	40.6	2.48	1.25	0.03	58.4	2.4
KK-N	63.7	0.51	74.7	2.68	1.17	0.02	53.4	3.20	0.84	0.03	30.6	1.9
Kap-N	7.8	0.41	9.5	0.77	1.21	0.14	10.1	0.31	1.28	0.01	9.5	0.4
AF23N	inactive		Inactive				inactive				>200	
CCN	9.1	0.17	10.8	0.80	1.19	0.10	8.7	0.20	0.95	0.19	32.7	0.95
BRN	6.9	0.30	7.2	0.28	1.04	0.58	NA				25.9	0.55
RSN	4.2	0.04	3.9	0.16	0.92	0.13	4.4	0.26	1.05	0.46	1.2	0.1
ION	5.9	0.02	6.0	0.21	1.01	0.73	5.2	0.10	0.88	0.00	17.2	0.75
UDN	12.1	0.32	12.6	0.08	1.04	0.35		NA			7.6	0.65
Pentamidine	0.0023	0.0002	0.5	0.04	225	<0.001	0.1	0.002	31.08	<0.001	18.3	1.9
Menadione	—	—	—	—	—	—	—	—	—	—	0.8	0.1

EC_50_ values are expressed in µM for pentamidine, and as µg/mL for the extracts, and represent the average and SEM of 3 or 4 experiments. ^1^n = 3; ^2^n = 4; ^3^unpaired two-tailed Student’s t-test comparing EC_50_ value of the resistant strain with that of the same sample for the control strain s427. RF: resistance factor, being the EC_50_ value for the resistant strain divided by the EC_50_ value for the control (sensitive) strain. ND = not determined.

### Profiling of Samples with HPLC-UV-ELSD

The chromatograms of the crude samples run on HPLC-UV-ELSD suggested a wide diversity in the chemical composition for the Nigerian samples. The samples could be divided into two groups, group **I** comprised of samples that were collected from the central part of Nigeria (AF2N3, P3N, KKN, S97N, S95N, KAPN, S96N) which mostly demonstrated an intense ELSD only response, with weak or absent of UV peaks suggesting a high content of terpenoids and/or fats, and the absence of any chromophore containing compounds such as flavonoids or lignans or other phenolic compounds. All of these samples were also noted to have at most weak activity against trypanosomes. In contrast, samples in group **II** were collected from Southern areas and showed strong UV-ELSD responses and high activity against *T*. *brucei*. Figure S1 shows a comparison of the chromatograms obtained for a Southern and a Central Nigerian sample.

### GC-MS Analysis of Samples

Profiling of the crude samples by GC-MS showed that group **I** samples demonstrated relatively similar chromatograms showing a group of intense peaks in the range of 41–43 min. The mass spectra of these peaks were searched against the NIST library and were identified as various triterpenoids, waxes and long chain fatty acids, with similarity scores of more than 700 which appeared to be the major chemical components in these samples and this explained the high responses in ELSD only. Table S1 shows some of the compounds identified by GC-MS in the group **I** samples according to matching against the NIST library. Most of the components in the group **I** samples had retention times >40 min.

In the case of group **II** samples, (from Rivers State, Nigerian) i.e. BRN and RSN, most of the GC-MS peaks eluted before 35 min, while for the ION sample the retention times were mainly less than 20 min. Figure S2 shows a comparison of the GC-MS chromatograms for three representative samples.

### LC-MS Analysis of Crude Extracts from Nigerian Propolis Samples

**Figure 2:**
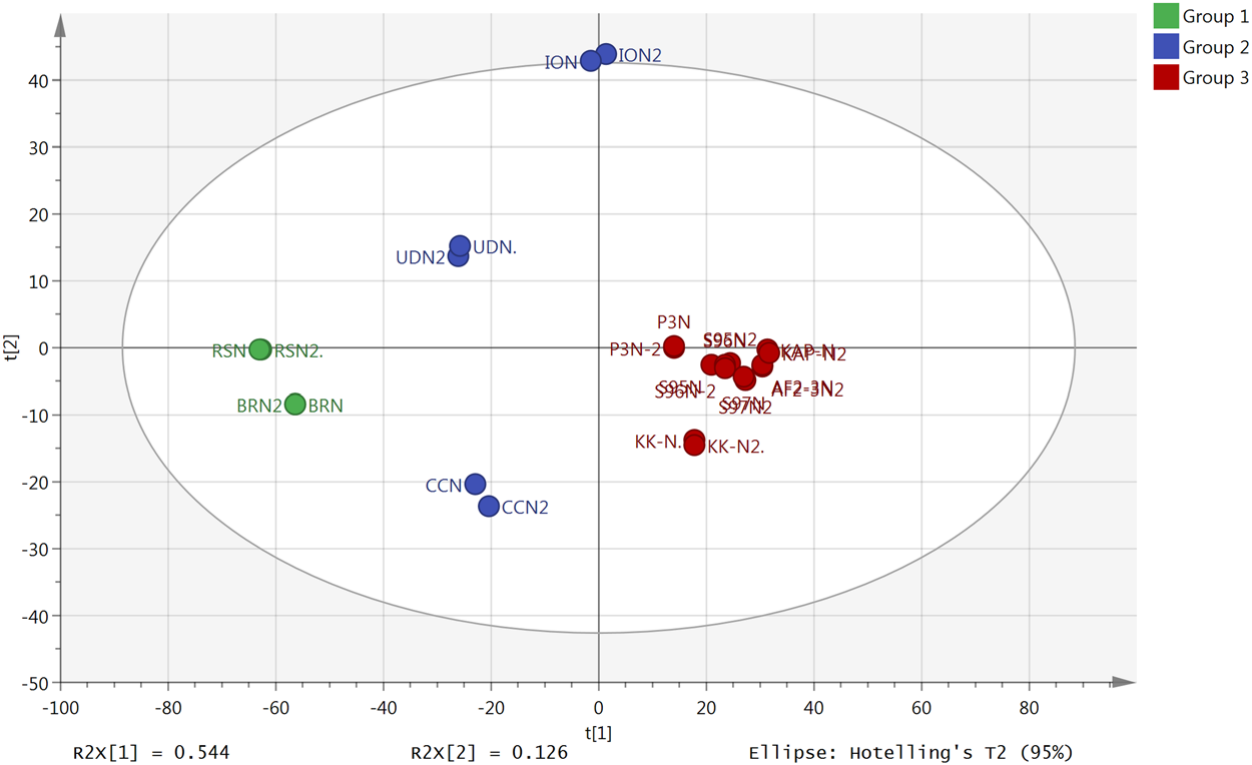
PCA model with HCA analysis for duplicate analyses of crude extracts of Nigerian propolis samples.

All samples were run in duplicate in order to verify the precision and reproducibility of the instrument. The data collected from the LC-MS were complex and difficult to process manually, and were split into positive and negative ion data, and then processed by MZ-mine 2.14^14^ as described recently by Siheri *et al*.^7^, and the extracted features were then searched against the Dictionary of Natural Products^15^ database. The 2000 most intense features with the highest mean peak areas across the 12 samples, selected by m/z mine from the negative ion data, were used to build a Principal Components Analysis (PCA) model (Fig. 2). The data was univariate scaled and log transformed prior to PCA modelling which was carried out by using Simca P 14.0. Hierarchical cluster analysis (HCA) was used to divide the samples into 3 groups. The score plot shows that most of the samples collected from central Nigeria are clustered near the centre due to the absence of LC–HRMS features with a high MS response. The compositions of the rest of samples, even for samples that were collected from the same area such as BRN and RSN (both of which were collected from Rivers State, Nigeria), were not identical. Samples UDN and CCN were also from the South of the country and were intermediate in composition between the samples from central Nigeria and those from Rivers State. The ION samples lay slightly outside the ellipse demonstrating a more unique chemical composition; from the loading plot characteristic chemical compounds responsible for variation among samples could be assigned exclusively to single chemical formulas within a 3 ppm mass error window. Each elemental composition generally corresponded to a large number of isomers within the DNP database.

### Structure elucidation of Compounds purified from Southern Nigeria propolis samples

Ten phenolic compounds were isolated and identified from the Rivers State samples (Coded BRN and RSN) in our previous work^6^.

LC-MS analysis of sample UDN showed several peaks in the extracted ion chromatogram for m/z 379.154 in negative ion ESI [M-H]^−^ corresponding to the formula C_23_H_24_O_5_. These components were targeted and isolation and to gave three compounds all of which were xanthones. Two of the xanthones, **1** and **2** (Figure S3) have been isolated previously from *Cudrania cochinchinensis* and *Cudrania tricuspidata* and the NMR spectra of compound **1** and **2** isolated from UDN matched the spectral data for gerontoxanthone H and 6-deoxv-γ-mangostin in literature^16, 17^. The mass spectral data and NMR data for **1** and **2** are given in S1, Tables S2 and S3.

### Compound 3

**Figure 3:**
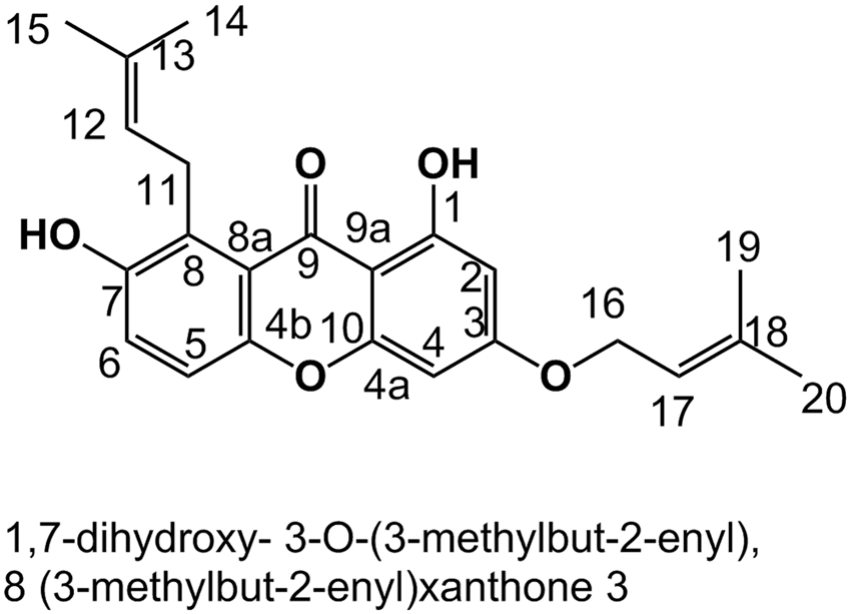
Structure of the new xanthone isolated from the UDN propolis sample.

The compound was obtained as a fine yellow powder (MP 179–181 °C). Analysis by LC-MS (rt 50.6 min) gave a molecular ion at m/z 379.1551 [M-H]^−^; calculated mass for C_23_H_23_O_5_ = 379.1545. Its ^1^H NMR spectrum (Table 3) shows a signal for a chelated hydroxyl group at δ_H_ 13.19 (1H, s). Four aromatic protons with AB spin systems were observed at 6.36 (1H, d, *J* = 2.3 Hz, H-2), 6.57 (1H, d, *J* = 2.3 Hz, H-4), 7.03 (1H, d, *J* = 8.2 Hz) and 7.23 (1H, d, *J* = 8.2 Hz). Two methylene protons were observed at δ 4.69 (2H, br d, *J* = 6.8 Hz, H-16) and at δ 3.90, (2H, br d, *J* = 7.2 Hz, H-11). Two methine protons were observed at δ 5.33 (m, H-12) and δ 5.47 (m, H-17). Four methyl groups appeared as doublets (*J* = 1.3) at δ 1.68, 1.71, 1.78, and 1.74. Analysis of its HMQC (^1^ *J*) and HMBC (^2^ *J* and ^3^ *J*) correlations enabled assignments of the proton and carbon signals as well as the positions of the two prenyl and two hydroxy groups as follows: correlations from the chelated –OH at C-1 identified C-1 (δ_C_ 162.5), C-2 (δ_C_ 97.9) and C-9a (δ_C_ 103.9). The methyl groups at δ_H_ 1.68 (H-14) and 1.71 (H-15) showed correlations to C-12 (δ_C_ 124.0) while the other two methyl groups at δ 1.78 (H-19) and 1.74 (H-20) showed correlations to C-17 (δ_C_ 119.6). The prenyloxy group must be at C-3 based on H-2, H-4 and H-16 correlations to C-3 and the non-oxygenated prenyl group must be at C-8 from correlations of H-12 and H-6 to C-8. Hence the two hydroxyl groups must be at C-1 and C-7. Comparing its NMR data to literature reports and that for calothwaiteaixanthone^18^ the compound was identified as 1,7-dihydroxy-8-(3-methylbut-2-enyl)-3-(methylbut-2-enyloxy) xanthone (Fig. 3). The structure was further confirmed by its mass ions obtained from HRESI-MS^n^ as follows: MS^2^ (% intensity) 310.0844 (100) C_18_H_15_O_5_ (-C_5_H_8_, loss of a prenyl group), MS^3^ 295.0612 (100) C_17_H_12_O_5_ (-CH_3_), 242.0220 (60) C_13_H_17_O_5_, 267.0299 (20) C_15_H_8_O_5_.

**Table 3:** ^1^H (400 MHz) and ^13^C NMR (100 MHz) data for compound 3 in ^2^H_6_ DMSO.

Position	Chemical shift δ ppm		HMBC
	^1^H (mult, *J* Hz)	^13^C (mult)	(^1^H-^13^C)
**1**	—	162.5 (C)	—
**2**	6.36 (d, 2.3)	97.9 (CH)	
**3**	—	156.8 (C)	—
**4**	6.57 (d, 2.3)	93.0 (CH)	C-2
**4a**	—	154.3 (C)	—
**4b**	—	146.4 (C)	—
**5**	7.03 (d, 8.2)	125.7 (CH)	C-7, C-8a
**6**	7.24 (d, 8.2)	120.7 (CH)	C-4b, C-8
**7**	—	144.5 (C)	—
**8**	—	132.9 (C)	—
**8a**	—	118.3 (C)	—
**9**	—	183.0 (C)	—
**9a**	—	103.9 (C)	—
**10**	—	—	—
**11**	3.90, 2H (d, 7.2)	32.8 (CH_2_)	C-8, C-12
**12**	5.33, 1H (t)	124.0 (CH)	
**13**	—	131.6 (C)	—
**14**	1.68, 3H (d, 1.3)	26.1 (CH_3_)	C-12, C-13, C-15
**15**	1.71, 3H (d, 1.3)	18.3 (CH_3_)	C-12, C-13, C-14
**16**	4.69, 2H (d, 6.8)	65.9 (CH_2_)	C-17, C-18
**17**	5.47, 1H (t)	119.6 (CH)	
**18**	—	138.7 (C)	—
**19**	1.78, 3H (d, 1.3)	25.9 (CH_3_)	C-17, C-18, C-20
**20**	1.74, 3H (d, 1.3)	18.6 (CH_3_)	C-17, C-18, C-19
**1-OH**	13.19, 1H (s)	—	C-1, C-2, C-9a
**7-OH**	10.21 (s)	—

Closely related xanthones were found for the first time in propolis collected by *Tetragonula laeviceps* stingless bees from Thailand. *Garcinia mangostana* (Mangosteen) was proposed as the most probable plant source^16^.

Fractionation of the ION sample resulted in the isolation of two cyclortanes ambonic **4** and mangiferonic acid **5** and a fraction containing α-amyrin **6** (Figure S4) with mangiferonic acid. The compounds were all identified by comparison with literature NMR data^19, 20^. The NMR data are summarised in Tables S4–S6. These kind of compounds were isolated before from Cameroonian propolis, and mango (*M*. *indica*, Anacardiaceae) seemed to be the plant source of the resin^19^. Mango is widely used in honey production in Cameroon and throughout tropical Africa^19^. α Amyrin and Mangiferonic were also found in Brazillian propolis^20^, and the latter has been isolated from Myanmar propolis and showed strong cytotoxic properties^21^.

### Bioassay results For the Isolated Compounds

Table 4 shows the bioassay results for the compounds isolated from the ION and UDN propolis samples. Only xanthone 1 was more active than the crude ION extract and the range of activity against *T*. *brucei* was dependent on the position of the prenyl substituent. Only α-amyrin was more active than the crude UDN sample. Similar to the crude samples, the purified compounds displayed only marginally different activities against the two drug resistant strains. Thus, the classes of compounds described herein would not be cross-resistant with the main drugs currently in use against human and animal African trypanosomiasis.

**Table 4:** Bioassay of compounds isolated from Nigerian propolis against *T*. *brucei* (n = 3).

Sample	T b S427WD		B48				aqp2/aqp3 null			
	AVG of EC50	SEM	AVG	SEM	RF	ttest	AVG	SEM	RF	ttest
Mangiferonic acid (5)	11.6	0.30	14.1	0.68	1.22	0.028	9.1	0.13	0.78	0.0014
Ambonic acid (4)	18.5	1.19	24.4	1.08	1.32	0.021	12.7	2.71	0.68	0.12
α-Amyrin (6)	8.9	0.88	10.6	0.81	1.20	0.22	8.2	0.23	0.93	0.52
Xanthone (1)	1.5	0.03	2.3	0.14	1.58	0.0042	0.8	0.02	0.54	<0.001
xanthone (2)	4.3	0.08	6.4	0.30	1.48	0.0026	4.3	0.05	0.99	0.75
xanthone (3)	5.6	0.20	6.8	0.39	1.21	0.054	6.4	0.22	1.15	0.051
Pentamidine	0.0023	0.0002	0.5	0.04	225	<0.001	0.1	0.002	31.1	<0.001

EC_50_ values are expressed in µM for pentamidine, and as µg/mL for the purified compounds, and represent the average and SEM of 3 independent experiments. Statistical significance was determined using an unpaired two-tailed Student’s t-test comparing EC_50_ value of the resistant strain with that of the same sample for the control strain s427. RF: resistance factor, being the EC_50_ value for the resistant strain divided by the EC50 value for the control (sensitive) strain.

### Prediction of the most active compounds in the crude propolis extracts from LC-MS data

**Figure 4:**
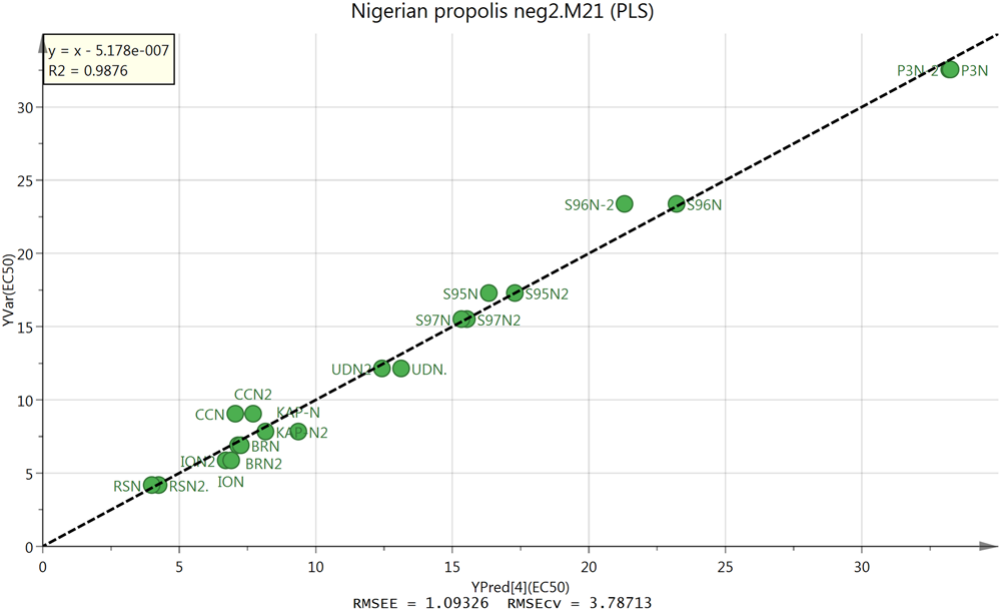
PLS model of predicted against measured anti-trypanosomal activity of Nigerian propolis extracts based on 180 features.

The extracted LC-MS data was used to build a Partial Least Squares (PLS) model for predicted against measured EC_50_ values against *T*. *brucei* (Fig. 4). Two of the low activity samples AF23N and KKN were omitted from the model. The model was edited to remove variables having less impact in the prediction of anti-trypanosomal activity and Fig. 4 is based on 180 features.

**Figure 5:**
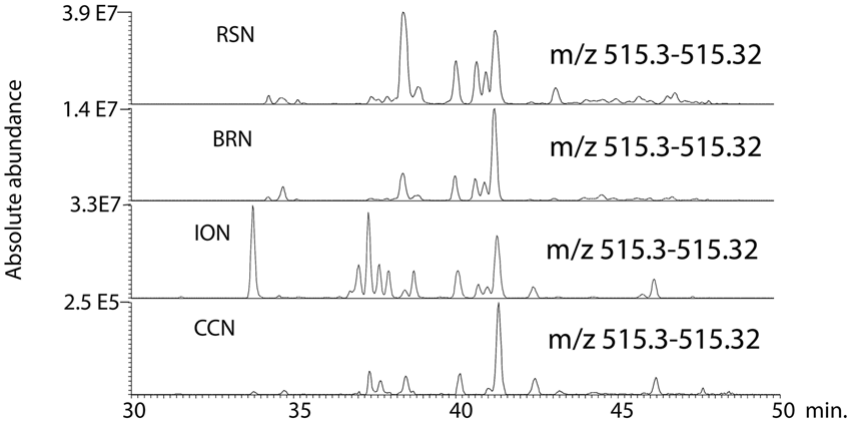
Extracted ion traces for denticulatain isomers in the high activity Nigerian propolis samples.

**Figure 6:**
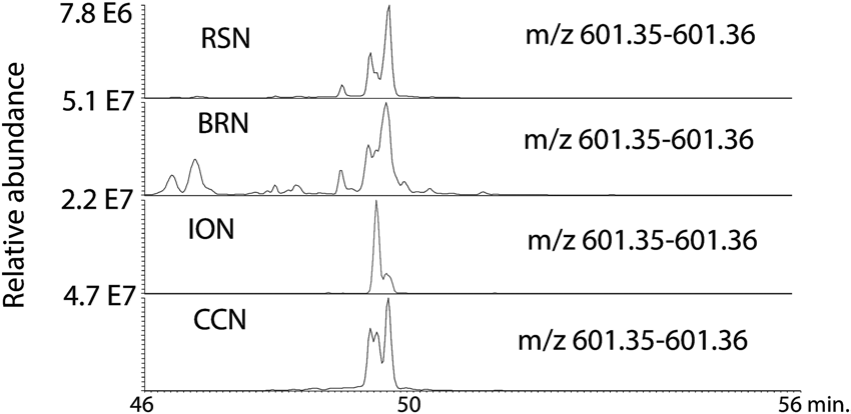
Extracted ion traces for isomers of guttiferone A samples RSN, BRN, ION and CCN.

When the loadings plot for the model (Figure S5) was examined it was clear that the compounds predicted to be most responsible for anti-trypanosomal were isomers of denticulatain, which we observed previously^6^. Most of the isomers had a characteristic fragment ion at m/z 241.05 as described in our previous paper^6^ where a detailed elucidation of the MS^2^ spectra is given. These compounds probably are obtained from *Macaranga* species^22^. Figure 5 shows an extracted ion trace for the denticulatain isomers in four high activity samples indicating that the abundance of these isomers correlates with activity against *T*. *brucei*. We were interested in the occurrence of guttiferone isomers, which we had observed in Rivers State propolis samples in our earlier study^5^ and speculated that these might be responsible for the high anti-trypanosomal activity of the of the samples since we had previously observed very high activity for a phloroglucinone compound isolated from Cameroonian propolis^23^. However, as can be seen in the extracted ion traces shown in Fig. 6 for guttiferone isomers sample CCN, which has lower anti-trypanosomal activity than sample.

RSN, contains much higher levels of these isomers than sample RSN. Thus it would appear that levels of denticulatain isomers correlate more closely with observed anti-trypanosomal activity. Thus these compounds should be targeted for isolation and testing.

## Discussion

The composition of Nigerian propolis samples varies widely according to geographical origin. Two main types were observed which were either rich in UV-absorbing phenolic compounds or contained mainly non-UV-absorbing triterpenoids. The highest anti-trypanosomal activities were displayed by the samples from southern Nigeria which had a high content of phenolic compounds. Six compounds were isolated from two of the Southern Nigerian samples comprised of three xanthones and three triterpenoids. The anti-trypanosomal acitivities of the isolated compounds in most cases were no higher than those of the crude extracts. In order to determine what the most active components in the extracts were PLS modelling was carried out using the high resolution mass spectrometry data for the extracts. The loadings plot for a PLS model pointed towards denticulatain isomers as being most strongly associated with the anti-trypansomal activities and thus a target for isolation and testing.

## Materials and Methods

### Materials

Absolute ethanol, HPLC grade acetonitrile, hexane, methanol, formic acid were obtained from Fisher Scientific (Loughborough, UK). Deuterated chloroform, dimethyl sulphoxide DMSO-d6, silica gel 60 (0.04–0.06 mm mesh size) and Wilmad nuclear magnetic resonance (NMR) tubes were obtained from Sigma Aldrich (Dorset, UK). AnalaR grade formic acid (98%) was obtained from BDH-Merck, UK. HPLC grade Water was produced in house by a Milli Q system (Millipore, UK). An ACE C18 column (3 mm × 150 mm, 3 μm) was purchased from Hichrom, (Reading, UK).

### Propolis sample collection and preparation

The propolis samples were either supplied by BeeVital (Whitby, UK) or collected by Dr John Igoli from various regions of Nigeria (Table 1, Fig. 1). All samples were sticky and brown in colour except samples collected in the Southern regions (RSN, BRN and UDN) which were reddish in colour. For profiling, 50 mg of each was extracted with ethanol (3 × 5 mL) by sonication at 40 °C for three hours each, and then maceration overnight with ethanol (5 mL). Each extract was filtered through filter paper, and then combined together, the filtered solutions were dried using a flow of nitrogen, the amount of residue was measured and the percentage yield was calculated. The ethanolic crude extracts were stored at −20 °C for further experiments.

### Profiling of the Extracts by ELSD and High Resolution Mass Spectrometry

For profiling experiments 2 mg of crude extract was reconstituted in 1 mL of methanol and filtered using a nylon syringe filter (Fisher Scientific, pore size 0.45 μm); 10 µL of the filtrate was injected into an Agilent 1100 HPLC system (Agilent Technologies, Germany) to a UV two channel detector (290 and 320 nm) coupled with an Evaporative Light Scattering Detector (ELSD) (model: SEDEX75, Sedere, France). The mobile phase used was water as mobile phase A and acetonitrile (ACN) as B at a flow rate of 0.3 mL/min. The gradient elution was programmed as follows: 0–15 min linear gradient from 30% to 50% of B, 15–25 min at 50% of B, 25–40 min linear gradient from 50% to 80% of B, 40–50 min at 80% of B, 50–51 min increasing to 100% of B, 51–59 min at 100% of B with the flow rate increasing to 0.5 mL/min and at 61 min back to 30% of B and hold until 70 min. The LC-HRMS analysis was performed on an Accela 600 HPLC system combined with an Exactive (Orbitrap) mass spectrometer from Thermo Fisher Scientific (Bremen, Germany) using the same conditions as for the UV-ELSD analysis but with the addition of 0.1% formic acid to mobile phases A and B. The samples were run in duplicate, the MS detection range was from m/z 100–1500 and scanning was performed under ESI polarity switching mode. The needle voltages were −4.0 kV, 4.5 kV positive and the sheath and auxiliary gases were set at 50 and 17 arbitrary units, respectively. The data obtained were split into positive and negative ions and the ‘negative’ dataset was processed using MZMine 2.14, with the masses selected between m/z 100–1200. In all experiments the column used was an ACE C18 column (150 × 3 mm, 3 µm particle size) (HiChrom, Reading UK). The Xcalibur 2.2 mass spectrometry data system from Thermo Fisher Scientific was used to check the data manually. For fragmentation of purified samples and extracts an LTQ-Orbitrap Classic mass spectrometer coupled with a Surveyor HPLC system from Thermo Fisher Scientific (Bremen, Germany) was employed in negative ion mode using collision-induced dissociation (CID) at 35 V. The same HPLC conditions were used as were used with the Exactive instrument. The mass axes of both MS instruments were externally calibrated according to the manufacturer’s instructions just before commencing the experiments.

### GC-MS Analysis

A portion of each extract (2 mg) was dissolved in 1 mL of ethyl acetate and 1 µL of each prepared sample was injected in splitless mode at 280 °C into the GC–MS (Focus GC-DSQ2, Thermo Fisher Scientific, Hemel Hempstead, UK) system equipped with a 30 m × 0.25 mm i.d., with 0.25 μm film thickness InertCap 1 MS capillary column from HiChrom, Reading UK. The temperature gradient was programmed as follows: 100 °C for 2 min, linearly increasing to 280 °C at the rate of 5 °C/min, holding at 280 °C for 15 mins and linearly increasing to 320 °C at the rate of 10 °C/min and holding for 10 mins. The source temperature was 250 °C and the ionisation voltage was 70 eV for EI–MS.

### Fractionation of Propolis samples

Two of the active samples coded ION, UDN were subjected to fractionation. 100 g of each sample was extracted with ethanol (500 mL) as described above and yielding 40 g and 61 g of ethanolic extract of ION and UDN respectively. Portions of the extracts ION (10 g) and UDN (7 g) were reconstituted with ethyl acetate (10 mL) mixed with (5 g) coarse silica, left in a fume hood to dry and were then loaded onto the top of a silica bed (50 g) in an open glass column (55 cm × 3 cm). The samples were then fractionated by using open column chromatography on silica gel, eluted with (2800 mL) of mobile phase with collection of 50 mL fractions as follows: 100% hexane to 100% ethyl acetate with 200 mL at each step stepwise as follows: 100% hexane-80:20–70:30–60:40–50:50–40:60–30:70–10:90–100% ethyl acetate and then continuing with increasing amounts of MeOH in EtOAc with 200 mL at each step as follows 10:90–20:80–30:70–50:50–60:40 resulting in 56 × 50 mL fractions.

In case of UDN two high weight fractions UDN10 (265 mg) collected at 40:60 EtOAc:hexane, and UDN14 (130 mg) eluted at 50:50 EtOAc:hexane, were chosen for further fractionation. The fractions were reconstituted in 5 mL EtOAc mixed with celite in a 1:2 proportion (v/v), dried and packed into an empty dryloader cartridge (Alltech, Carnforth, UK) to be transferred to a Grace Davison Reveleris® flash chromatography system (Alltech, Carnforth, UK) equipped with a dual-UV wavelength detector that was set at 290 and 320 nm, an ELSD, and an automatic fraction collector which collected peaks according to the threshold set for the ELSD (set to medium). After method development with an analytical LC-UV-ELSD system. Both open column fractions were re-chromatographed using the Grace system in reversed phase mode using a (12 g) C18 cartridge and a flow rate of 12 mL/min and isocratic conditions with 30:70 ACN:water for 30 min then increasing to 100% ACN over 30 min and holding for 5 min then back to 30% ACN in 1 min and holding for 5 min. This yielded compounds **1** (8.6 mg), **2** (11.0 mg) and **3** (7.0 mg); all were obtained as fine yellow powders.

In the case of the open column fractions of the ION sample, fractions ION12 (165 mg) and ION13 (160 mg) both eluted at 60:40 hexane:EtOAc. They were purified by using the reversed phase C18 (12 g) cartridge. The elution method used isocratic conditions with 90:10 ACN:water for 30 min with a flow rate of 9 mL/min yielding compounds **4** (13 mg), **5** (6 mg) and **6** (12.2 mg as a 3:1 mixture with 5) which were all white amorphous solids.

### NMR Spectra

The ^1^H, ^13^C and DEPT 135, and 2D ^1^H, ^1^H-COSY, and ^13^C-^1^H HSQC and HMBC NMR spectra were obtained by a JEOL-LA 400 FT-NMR spectrometer system using C^2^HCl_3_ and ^2^H_6_-DMSO as solvents.

### Anti-trypanosomal testing

All crude extracts and purified compounds were screened using a variant of the Alamar blue assay^24, 25^, based on the reduction of the blue dye resazurin sodium salt (Sigma-Aldrich) to fluorescent and pink resorufin by live trypanosomes^26^. In this screen testing was carried out against the standard drug-sensitive *T*. *b*. *brucei* clone and two derived drug resistant lines, in order to assess the potential for cross-resistance with existing drugs. The results were expressed as half maximal effective concentration (EC_50_) values, each based on at least three independent determinations. The assays were performed using serial dilutions in white opaque plastic 96-well plates (F Cell Star, Greiner Bio-one GmbH, Frickenhausen, Germany), with each compound or mixture double diluted over two rows of the plate (i.e. 23 double dilutions and a no-drug control well), allowing an optimally defined EC_50_ value after plotting of the fluorescence intensity against test compounds concentration and fitting to a sigmoid curve with variable slope (GraphPad Prism 5.0). Bloodstream forms of the following clonal strains of *T*. *b*. *brucei* were utilised: Lister strain 427 (s427)^27^, the standard drug-sensitive control strain; the B48 clone that was derived by *in vitro* adaptation of s427 to pentamidine^28^; and the aqp2/aqp3 null strain^13^, from which the gene encoding the High Affinity Pentamidine Transporter (HAPT1) has been deleted. For each strain, the seeding density at the start of the assay was 2 × 10^4^ cells/well, and the cells were exposed for 48 h to the test compounds, at 37 °C/5% CO_2_, before the addition of the resazurin dye and a further incubation of 24 h under the same conditions. Fluorescence was determined in a FLUOstar Optima (BMG Labtech, Aylesbury, UK) at wavelengths of 544 nm and 620 nm for excitation and emission, respectively.

All crude extracts were also tested against *C*. *fasciculata* (ATCC 50083) as described in our previous publication^7^.

## Electronic supplementary material

Supplementary material

## References

[1] Ristivojević P, Trifković J, Andrić F, Milojković-Opsenica D (2015). Poplar-type Propolis: Chemical Composition, Botanical Origin and Biological Activity. Nat. Prod. Communications.

[2] Banskota AH, Tezuka Y, Kadota S (2001). Recent progress in pharmacological research of propolis. Phytotherapy Research.

[3] Bankova V (2005). Chemical diversity of propolis and the problem of standardization. Journal of Ethnopharmacology.

[4] Araújo AS (2005). Electrospray ionization mass spectrometry fingerprinting of beer. Analyst.

[5] Zhang T (2014). Chromatographic analysis with different detectors in the chemical characterisation and dereplication of African propolis. Talanta.

[6] Omar RM (2016). Chemical characterisation of Nigerian red propolis and its biological activity against. Trypanosoma Brucei. Phytochemical Analysis.

[7] Siheri W (2016). Chemical and Antimicrobial Profiling of Propolis from Different Regions within Libya. PloS One.

[8] Giordani F, Morrison LJ, Rowan TG, DE KONING HP, BARRETT MP (2016). The animal trypanosomiases and their chemotherapy: a review. Parasitology.

[9] Graf FE (2015). Chimerization at the AQP2–AQP3 locus is the genetic basis of melarsoprol–pentamidine cross-resistance in clinical *Trypanosoma brucei* gambiense isolates. International Journal for Parasitology: Drugs and Drug Resistance.

[10] Trouiller P (2002). Drug development for neglected diseases: a deficient market and a public-health policy failure. The Lancet.

[11] Schlüns H, Sadd BM, Schmid-Hempel P, Crozier RH (2010). Infection with the trypanosome *Crithidia bombi* and expression of immune-related genes in the bumblebee *Bombus terrestris*. Developmental & Comparative Immunology.

[12] Ravoet J (2013). Comprehensive bee pathogen screening in Belgium reveals *Crithidia mellificae* as a new contributory factor to winter mortality. PLoS One.

[13] Baker N (2012). Aquaglyceroporin 2 controls susceptibility to melarsoprol and pentamidine in African trypanosomes. Proceedings of the National Academy of Sciences.

[14] Pluskal T, Castillo S, Villar-Briones A, Orešič M (2010). MZmine 2: modular framework for processing, visualizing, and analyzing mass spectrometry-based molecular profile data. BMC Bioinformatics.

[15] Buckingham J. Dictionary of Natural Products. CRC Press ISBN 9780412466205 (1993).

[16] Sanpa S (2015). Antibacterial compounds from propolis of *Tetragonula laeviceps* and *Tetrigona melanoleuca* (Hymenoptera: Apidae) from Thailand. PloS One.

[17] Chang C-H, Lin C-C, Kawata Y, Hattori M, Namba T (1989). Prenylated xanthones from *Cudrania cochinchinensis*. Phytochemistry.

[18] Bandara BR, Ranjith H, Dharmaratne W, Sotheeswaran S, Balasubramaniam S (1986). Two chemically distinct groups of Calophyllum species from Sri Lanka. Phytochemistry.

[19] Kardar M (2014). Characterisation of triterpenes and new phenolic lipids in Cameroonian propolis. Phytochemistry.

[20] Silva MdSSd, Citó AMdGL, Chaves MH, Lopes JAD (2005). Cycloartane triterpenoids of propolis from Teresina-PI. Química Nova.

[21] Li F (2009). Chemical constituents of propolis from Myanmar and their preferential cytotoxicity against a human pancreatic cancer cell line. Journal of Natural Products.

[22] Yang D-S (2015). Denticulatains A and B: unique stilbene–diterpene heterodimers from *Macaranga denticulata*. RSC Advances.

[23] Almutairi S (2014). New anti-trypanosomal active prenylated compounds from African propolis. Phytochemistry Letters.

[24] Wallace LJ, Candlish D, De Koning HP (2002). Different substrate recognition motifs of human and trypanosome nucleobase transporters selective uptake of purine antimetabolites. Journal of Biological Chemistry.

[25] Rodenko B (2007). 2, N6-disubstituted adenosine analogs with antitrypanosomal and antimalarial activities. Antimicrobial Agents and Chemotherapy.

[26] de Koning HP, MacLeod A, Barrett MP, Cover B, Jarvis SM (2000). Further evidence for a link between melarsoprol resistance and P2 transporter function in African trypanosomes. Molecular and Biochemical Parasitology.

[27] Gould MK, Vu XL, Seebeck T, de Koning HP (2008). Propidium iodide-based methods for monitoring drug action in the kinetoplastidae: comparison with the Alamar Blue assay. Analytical biochemistry.

[28] Bridges DJ (2007). Loss of the high-affinity pentamidine transporter is responsible for high levels of cross-resistance between arsenical and diamidine drugs in African trypanosomes. Molecular Pharmacology.

