# Supplementary material for: The Chemical Characterization of Nigerian Propolis samples and Their Activity Against Trypanosoma brucei
Source: Sci Rep. 2017 Apr 19;7:923. doi: 10.1038/s41598-017-01038-2 (PMC5430459; doi:10.1038/s41598-017-01038-2)

The Chemical Characterization of Nigerian Propolis samples and Their Activity Against *Trypanosoma brucei*

Ruwida Omar[1], John Igoli[1,2], Tong Zhang[3] ,Alexander I. Gray[1], Godwin U Ebiloma[4], Carol J Clements[1], James Fearnley[5], RuAngeli Edrada Ebel[1], Tim Paget[6], Harry P. De Koning[4] and *David G. Watson[1].

1. University of Strathclyde, Strathclyde Institute of Pharmacy and Biomedical Science, 161 Cathedral Street, Glasgow, G4 0RE, UK.
2. Phytochemistry Research Group, Department of Chemistry, University of Agriculture Makurdi, Nigeria.
3. Wolfson Wohl Cancer Research Centre, Institute of Cancer Sciences, University of Glasgow, Switchback Road, Glasgow, UK G61 1QH.
4. Institute of Infection, Immunity and Inflammation, College of Medical, Veterinary and Life Sciences, University of Glasgow, Glasgow G12 8TA, UK.
5. BeeVital, Whitby, North Yorkshire, YO22 5JR, UK.
6. Dept. of Pharmacy, Health and Well-being, University of Sunderland, Wharncliffe Street Sunderland SR1 3SD.

Table S1. Some compounds identified in the group I Nigerian propolis samples by GC-MS.

compound	Formula	Retention time(min.)
Ursenol	$C_{30}H_{50}O$	41.2
Cholestan-3ol-2methylene	$C_{28}H_{48}O$	41.5
Lupeol	$C_{30}H_{50}O$	43.5
α and β-Amyrin	$C_{30}H_{50}O$	43.6
Lupenol acetate	$C_{32}H_{52}O_2$	43.9

Table S2. NMR sand MS data for compound **1** (1,3,7-trihydroxy-2,8-di-(3-methylbut-2-enyl)xanthone)

Fine yellow powder ; HR-EIMS Rt 47.8 min m/z (% rel. int.)379.1544(100) [M-H]$^-$ calculated for $C_{23}H_{24}O_5$, MS^2 356.0894(40) $C_{19}H_{17}O_7$,ppm -2.05, 323.0920 (30) $C_{19}H_{16}O_5$,ppm -1.35, 281.0452 (30) $C_{16}H_{10}O_5$,ppm -1.13, 335.0918 (20) $C_{20}H_{16}O_5$,ppm -2.08, 309.0764(10) $C_{18}H_{14}O_5$,ppm -1.41; 1H NMR (DMSO, 400 MHz) and ^{13}C NMR (DMSO, 100 MHz).

**Position**	^{1}H δ ppm (mult, *J*)	^{13}C δ ppm
**1**	-	160.0(C)
**2**	-	110.1(C)
**3**	-	163.3(C)
**4**	6.48, 1H(s)	93.0(CH)
**4a**	-	154.7(C)
**4b**	-	146.3(C)
**5**	6.99, 1H(d,8.2)	125.5(CH)
**6**	7.20,1H(d,8.2)	120.3(CH)
**7**	-	144.6(C)
**8**	-	132.5(C)
**8a**	-	118.6(C)
**9**	-	191.1(C)
**9a**	-	102.5(C)
**10**	-	-
**11**	3.89, 2H(d,7.2)	33.1(CH_2)
**12**	5.32,1H(br.t)	124.2(CH)
**13**	-	131.4 (C)
**14**	1.70, 3H(s)	18.3(CH_3)
**15**	1.68, 3H(s)	26.0(CH_3)
**16**	3.25, 2H(d, 8.0)	21.6(CH_2)
**17**	5.19,1H(br.t)	122.9(CH)
**18**	-	130.7(C)
**19**	1.64, 3H (s)	26.1(CH_3)
**20**	1.75, 3H(s)	18.3(CH_3)
**1-OH**	13.51, 1H(s)	-

Table S3. NMR and MS data for compound 2 (1,3,7-trihydroxy-4,8-di-(3-methylbut-2enyl)xanthone)

Fine yellow powder ; HR-EIMS Rt 41.9 min m/z (% rel. int.)379.1550[M-H]$^-$(100) calculated for $C_{23}H_{24}O_5$, MS^2 356.0904 (60) $C_{19}H_{17}O_7$,ppm 0.5, 324.1005 (40) $C_{19}H_{17}O_5$,ppm 0.4, 310.0847 (30) $C_{18}H_{15}O_5$,ppm 0.155, 267.0300 (20) $C_{15}H_8O_5$,ppm 0.5, 255.0300(20) $C_{14}H_7O_5$,ppm 0.3; 1H NMR (DMSO, 400 MHz) and and ^{13}C NMR (DMSO, 100 MHz).

Position	1H δ ppm (mult, J)	13C δ ppm
**1**	-	161.0 (C)
**2**	6.27,1H(s)	97.6(CH)
**3**	-	163.2 (C)
**4**	-	106.0 (C)
**4a**	-	153.7 (C)
**4b**	-	146.8 (C)
**5**	6.99,1H(d,8.2)	125.4(CH)
**6**	7.21,1H(d, 8.2)	120.5(CH)
**7**	-	145.0 (C)
**8**	-	132.7 (C)
**8a**	-	118.3 (C)
**9**	-	190.9 (C)
**9a**	-	103.2 (C)
**10**	-	-
**11**	3.89 ,2H(d,7.1)	33.04(CH2)
**12**	5.31,1H(m)	124.1 (CH)
**13**	-	131.9 (C)
**14**	1.70, 3H(s)	18.3(CH3)
**15**	1.67, 3H(s)	26.08(CH3)
**16**	3.45,2H(d,7.1)	21.53(CH2)
**17**	5.29 ,1H(m)	122.67(CH)
**18**	-	133.4 (C)
**19**	1.80,3H(s)	18.8(CH3)
**20**	1.62,3H(s)	26.5(CH3)
**1-OH**	13.18,1H(s)	-
**3-OH**	10.92,1H(s)	-
**7-OH**	10.01,1H(s)	-

Table S4. NMR and MS data for compound 4 ambonic acid.

White amorphous solid, $[M\text{-}H]^-$ m/z 467.3531(calculated for $C_{31}H_{48}O_3$)1H NMR (CDCl3, 400 MHz) and ^{13}C NMR (CDCl3, 100 MHz).

**position**	^1H δ ppm (mult, *J*)	^{13}C δ ppm
**1**	1.88 ,1.57,2H,(m)	33.4(CH_2)
**2**	2.34(ddt, 14.0, 4.4, 2.6)<br>2.74(td, 13.9, 6.4)	37.5(CH_2)
**3**	-	219.6(C)
**4**	-	50.2(C)
**5**	1.74,1H(dd,12.3,4.5)	48.4(CH)
**6**	1.57,1.61,2H(m)	21.5(CH_2)
**7**	1.95,2H(m)	28.1(CH_2)
**8**	1.62,1H(m)	47.9(CH)
**9**	-	21.05(C)
**10**	-	25.9(C)
**11**	1.40, 2H(m)	25.9(CH_2)
**12**	1.69,2H(m)	32.8(CH_2)
**13**	-	45.3(C)
**14**	-	48.7(C)
**15**	1.35,2H(m)	35.6(CH_2)
**16**	1.19, 1.22,2H(m)	26.7(CH_2)
**17**	1.64,1H(m)	52.3(CH)
**18**	1.02,3H(s)	18.1(CH_3)
**19**	0.82,1H(d,4.2)<br>0.60,1H(d,4.3)	29.5(CH_2)
**20**	1.46,1H(m)	36.0(CH)

**21**	0.93,3H(d,7.1)	18.3(CH_3)
**22**	1.65,1.20, 2H(m)	34.7(CH_2)
**23**	2.25,1H (ddd, 15.7, 11.4, 4.8)<br>2.08,1H t(7.8)	31.6(CH_2)
**24**	-	148.5(C)
**25**	3.21, 1H(q,7.0)	45.4(CH)
**26**	-	178(C)
**27**	1.35,3H(d,7.0)	16.4(CH_3)
**28**	1.13,3H(s)	20.8(CH_3)
**29**	1.08,3H(s)	22.2(CH_3)
**30**	1.03,3H(s)	18.3(CH_3)
**31**	4.9,1H(brs)<br>5.0,1H(brs)	111.1(CH_2)

Table S5. NMR and MS data for compound 5 15-Mangiferonic acid.

White amorphous solid, $[M-H]^-$ m/z 453.3378(calculated for $C_{30}H_{46}O_3$), 1H NMR (CDCl3, 400 MHz) and ^{13}C NMR ($CDCl_3$, 100 MHz).

**Position**	^1H δ ppm (mult, *J*)	^{13}C δ ppm
**1**	1.88,1.57,2H,(m)	33.4(CH_2)
**2**	2.72,1H(ddd,13.9,6.4)<br>2.32,1H(ddd,14.1,4.4,2.6)	37.4(CH_2)
**3**	-	216.7(C)
**4**	-	50.2(C)
**5**	1.72 m	48.4(CH)
**6**	1.56 m, 1.65m	21.4(CH_2)
**7**		25.9(CH_2)
**8**	1.58,1H(m)	47.8(CH)
**9**	-	21.0(C)
**10**	-	25.9(C)
**11**	2.05,2H(m)	26.6(CH_2)
**12**	1.66, 2H(m)	32.7(CH_2)
**13**	-	45.4(C)
**14**	-	48.7(C)
**15**	1.32,2H(m)	35.5(CH_2)
**16**	1.92,2H(m)	28.1(CH_2)
**17**	1.61,1H(m)	52.2(CH)
**18**	1.01,3H (*s*)	18.1(CH_3)
**19**	0.58,1H (d,4)<br>0.80,1H (d,4)	29.5(CH_2)
**20**	1.46,1H(m)	35.9(CH)
**21**	1.01,3H(d,2.13)	18.1(CH_3)

**22**	1.17,2H (m)	34.7(CH_2)
**23**	2.15,2H(m)	25.8(CH_2)
**24**	6.90,1H(td,7.5,1.55)	145.7(CH)
**25**	-	126.7(C)
**26**	-	171.9(C)
**27**	1.86,3H(*s*)	12.0(CH_3)
**28**	1.06,3H(*s*)	22.1(CH_3)
**29**	1.11,3H(*s*)	20.8(CH_3)
**30**	0.92,3H(*s*)	19.2(CH_3)

Table S6. NMR and MS data for compound 6 α amyrin.

White amorphous solid, $[M\text{-}H]^-$ m/z 425.2460 (calculated for $C_{30}H_{50}O_3$), 1H NMR (CDCl3, 400 MHz) and ^{13}C NMR (CDCl3, 100 MHz). The NMR data is shown in table S5 and correlates with the NMR spectra published previously.

**Position**	^1H δ ppm (mult, *J*)	^{13}C δ ppm
**1**		38.9(CH_2)
**2**		27.5(CH_2)
**3**	3.23,1H (*dd*, 4.7, 11.5)	78.8(CH)
**4**	-	38.8(C)
**5**	0.75,1H(d,11.7)	55.7(CH)
**6**	1.46,1.55,2H(m)	18.3(CH_2)
**7**	1.69,1.70,2H(m)	32.78(CH_2)
**8**	-	40.36(C)
**9**	1.39,1H(s)	50.5(CH)
**10**	-	37.5(C)
**11**	2.06	23.01(CH_2)
**12**	5.14,1H (t, 3.6)	124.7(CH)
**13**	-	140.6(C)
**14**	-	49.3(C)
**15**	1.5, 2H(m)	28.15(CH_2)
**16**	2.06,2H(m)	26.9(CH_2)
**17**	-	33.4(C)
**18**	1.63,1H(m)	52.23(CH)
**19**		39.15
**20**		39.21
**21**	1.66,2H(m)	32.8(CH_2)
**22**	1.512H(m)	40.5(CH_2)

**23**	1.00,3H(s)	28.0(CH_3)
**24**	0.80 ,3H(s)	15.4(CH_3)
**25**	0.87 ,3H(s)	15.5(CH_3)
**26**	1.07 ,3H(s)	15.8(CH_3)
**27**	1.03,3H(s)	26.0(CH_3)
**28**	0.94 ,3H(s)	28.1(CH_3)
**29**	1.03 ,3H(s)	33.3(CH_3)
**30**	1.13 ,3H(s)	20.8(CH_3)

Figure S1. ELSD-UV chromatograms of two samples AF2-3N and ION from Central and Southern Nigeria.

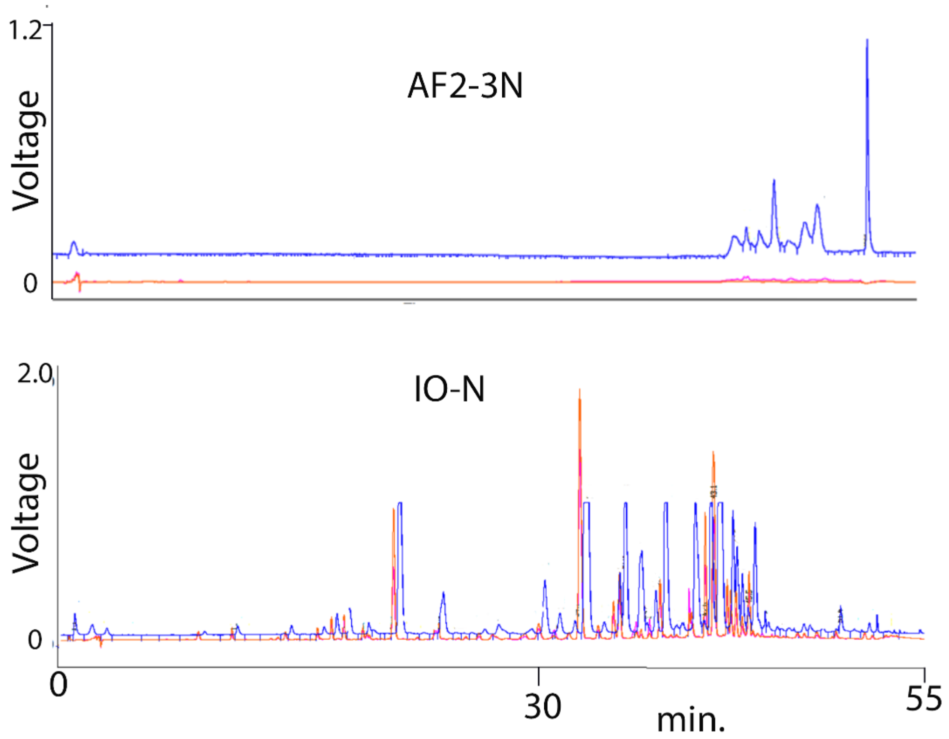

Figure S2 Representative GC–MS chromatograms for three propolis samples from different regions.

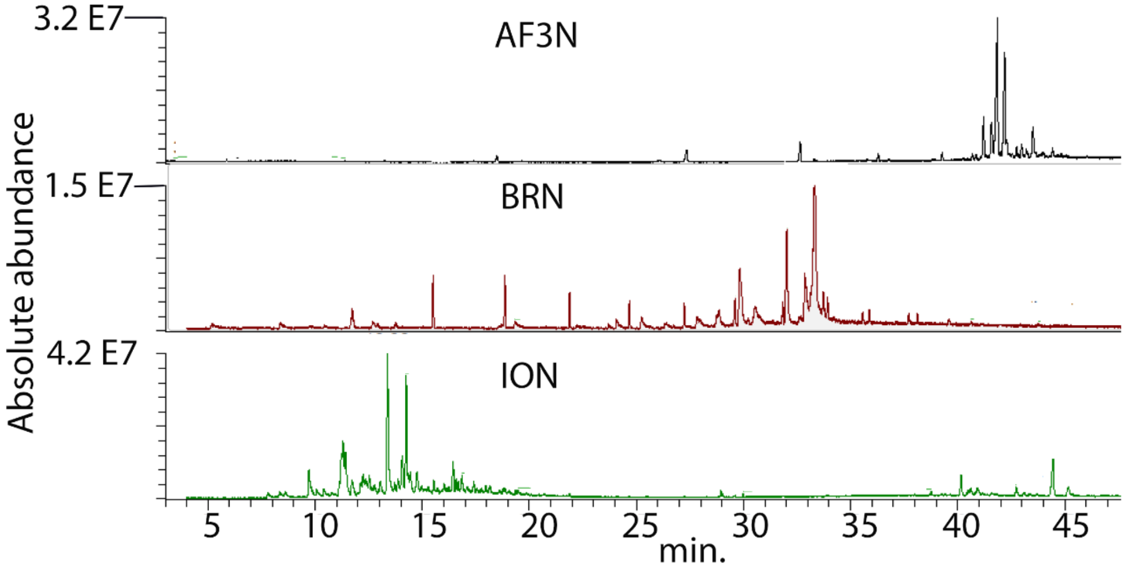

Figure S3 Known xanthones isolated from propolis sample UDN.

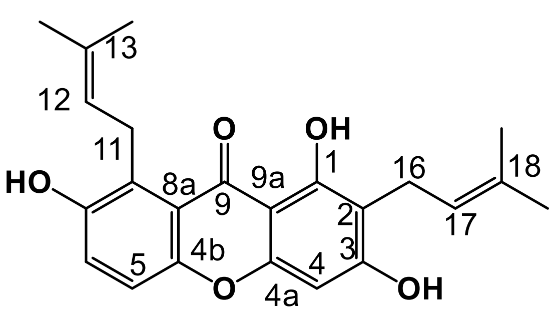

1,3,7-trihydroxy-2,8-di-
(3-methylbut-2-enyl)xanthone **(1)**

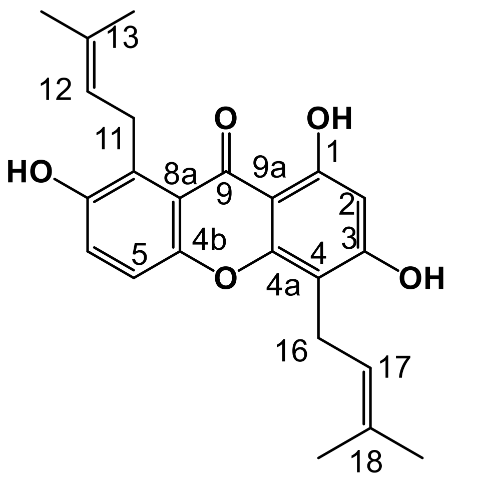

1,3,7-trihydroxy-4,8-di-
(3-methylbut-2enyl)xanthone **(2)**

Figure S4. Structures of the triterpenes isolated from the ION propolis sample.

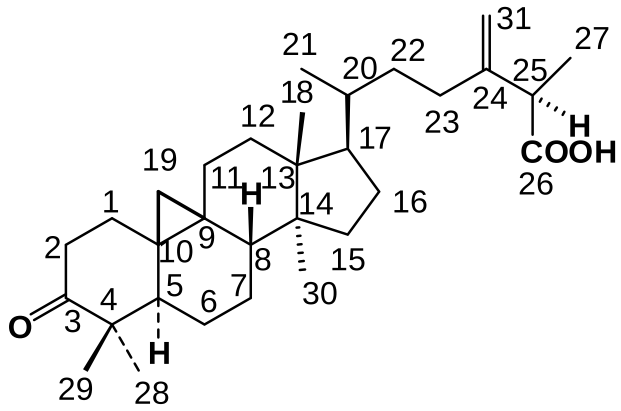

14-Ambonic acid **(4)**

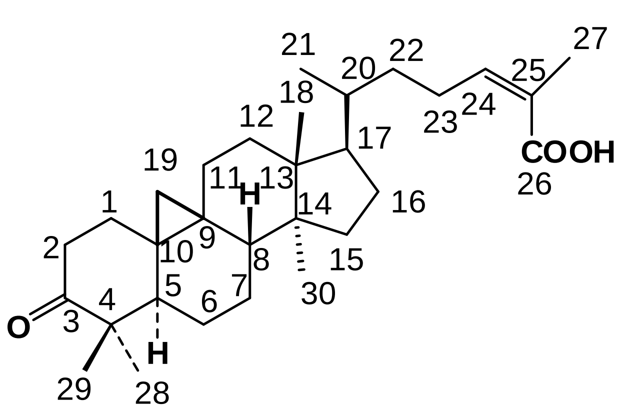

15-mangiferonic acid **(5)**

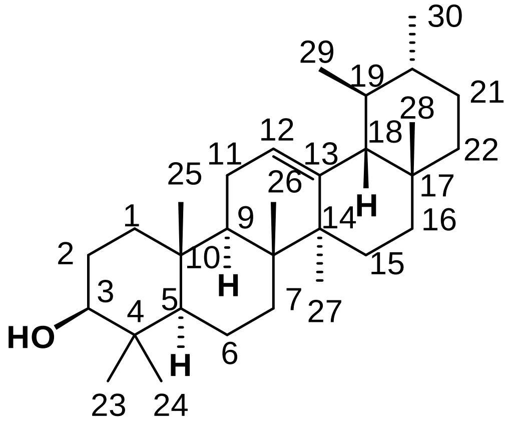

16-α-amyrin **(6)**

Figure S5. Loadings of the PLS plot in the high activity region for the Nigerian propolis samples.

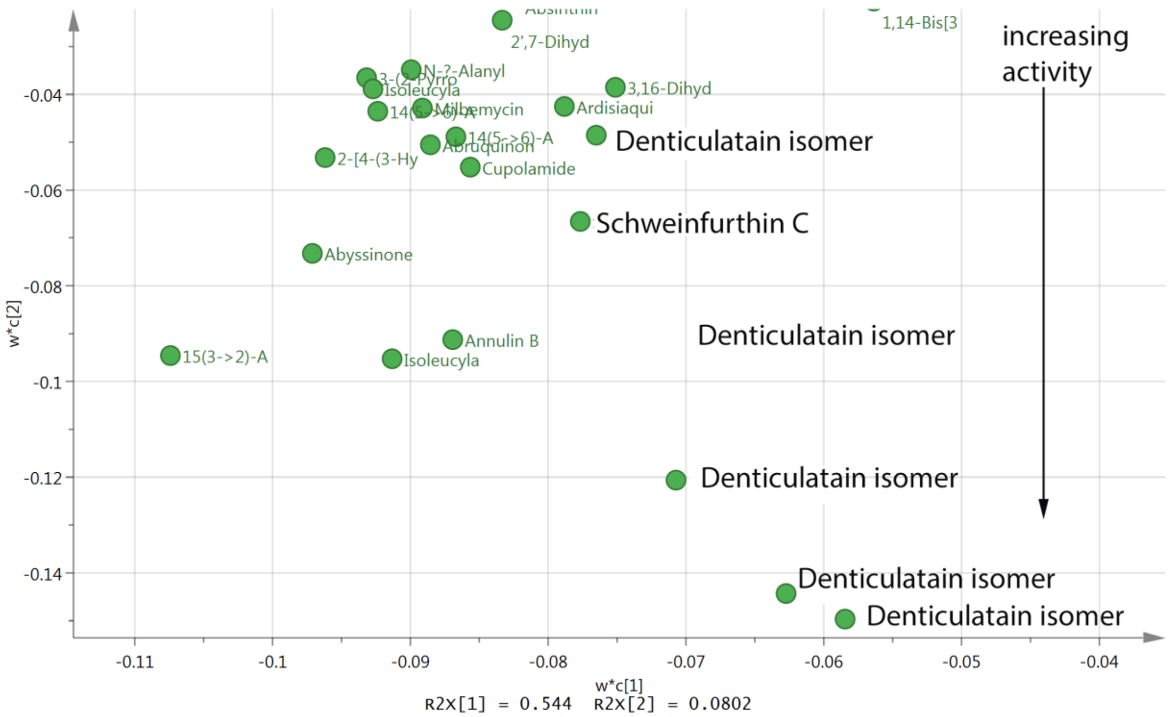

Supplement: Supplementary file 1 — Supplementary material [file 41598_2017_1038_MOESM1_ESM.pdf]
